# Drug-Resistant Tuberculosis Hotspots in Oliver Reginald Tambo District Municipality, Eastern Cape, South Africa

**DOI:** 10.3390/idr16060095

**Published:** 2024-12-06

**Authors:** Lindiwe Modest Faye, Mojisola Clara Hosu, Teke Apalata

**Affiliations:** Department of Laboratory Medicine and Pathology, Walter Sisulu University, Private Bag X5117, Mthatha 5099, South Africa; mhosu@wsu.ac.za (M.C.H.); tapalata@wsu.ac.za (T.A.)

**Keywords:** DR-TB, O.R. Tambo District Municipality, TB hotspots, sociodemographic factors, modeling, linear regression, random forest and tuberculosis case trends

## Abstract

Background: The global push to eliminate tuberculosis (TB) as a public health threat is increasingly urgent, particularly in high-burden areas like the Oliver Reginald Tambo District Municipality, South Africa. Drug-resistant TB (DR-TB) poses a significant challenge to TB control efforts and is a leading cause of TB-related deaths. This study aimed to assess DR-TB transmission patterns and predict future cases using geospatial and predictive modeling techniques. Methods: A retrospective cross-sectional study was conducted across five decentralized DR-TB facilities in the O.R. Tambo District Municipality from January 2018 to December 2020. Data were obtained from Statistics South Africa, and patient GPS coordinates were used to identify clusters of DR-TB cases via DBSCAN clustering. Hotspot analysis (Getis-Ord Gi) was performed, and two predictive models (Linear Regression and Random Forest) were developed to estimate future DR-TB cases. Analyses were conducted using Python 3.8 and R 4.1.1, with significance set at *p* < 0.05. Results: A total of 456 patients with DR-TB were enrolled, with 56.1% males and 43.9% females. The mean age was 37.5 (±14.9) years. The incidence of DR-TB was 11.89 cases per 100,000 population, with males being disproportionately affected. Key risk factors included poverty, lack of education, and occupational exposure. The DR-TB types included RR-TB (60%), MDR-TB (30%), Pre-XDR-TB (5%), XDR-TB (3%), and INHR-TB (2%). Spatial analysis revealed significant clustering in socio-economically disadvantaged areas. A major cluster was identified, along with a distinct outlier. The analyses of DR-TB case trends using historical data (2018–2021) and projections (2022–2026) from Linear Regression and Random Forest models reveal historical data with a sharp decline in DR-TB case, from 186 in 2018 to 15 in 2021, highlighting substantial progress. The Linear Regression model predicts a continued decline to zero cases by 2026, with an R^2^ = 0.865, a mean squared error (MSE) of 507.175, and a mean absolute error (MAE) of 18.65. Conversely, the Random Forest model forecasts stabilization to around 30–50 cases annually after 2021, achieving an R^2^ = 0.882, an MSE of 443.226, and an MAE of 19.03. These models underscore the importance of adaptive strategies to sustain progress and avoid plateauing in DR-TB reduction efforts. Conclusions: This study highlights the need for targeted interventions in vulnerable populations to curb DR-TB transmission and improve treatment outcomes.

## 1. Introduction

Tuberculosis (TB) remains a significant public health challenge globally, particularly in regions with high human immunodeficiency virus (HIV) prevalence, such as South Africa [1]. Among the various forms of TB, drug-resistant tuberculosis (DR-TB) presents an especially formidable challenge due to its resistance to standard first-line anti-TB drugs, complicating treatment regimens and reducing the chances of successful outcomes [2]. DR-TB arises primarily from two mechanisms: namely, primary drug resistance, which occurs through person-to-person transmission of resistant strains, and secondary drug resistance, which develops during treatment when patients do not adhere to their prescribed regimens or when inappropriate medications are used [3,4,5]. There are several types of drug-resistant TB diseases: namely, mono-resistant TB (resistant to one TB drug: rifampicin-resistant tuberculosis (RR-TB) resistant to rifampicin but susceptible to isoniazid, and isoniazid-resistant tuberculosis (INH-R TB), which is resistant to isoniazid but susceptible to rifampicin), poly-resistant TB (resistant to at least two TB drugs, but not both isoniazid and rifampin), multidrug-resistant TB (MDR TB, resistant to at least isoniazid and rifampin), pre-extensively drug-resistant TB (Pre-XDR TB, a type of MDR TB resistant to isoniazid, rifampin, and either a fluoroquinolone or a second-line injectable), and extensively drug-resistant TB (XDR TB, a rare type of MDR TB resistant to isoniazid, rifampin, a fluoroquinolone, and either a second-line injectable or additional drugs like bedaquiline or linezolid) [2,6]. DR-TB is caused by various factors including inadequate treatment, biological mutations, healthcare system challenges, socio-economic conditions, and HIV co-infection [7,8]. Poor treatment adherence, stigma (categorized into perceived stigma, which refers to the fear of negative judgment from others, and internalized stigma, where individuals accept negative stereotypes about themselves), and cultural beliefs can increase the risk of resistance [9]. HIV patients are more vulnerable due to weakened immune systems, making treatment harder and increasing the risk of drug-resistant TB [10]. Proper management can prevent drug resistance.

The global strategy to end the global TB epidemic by 2030 demands an imperative action plan by all countries that are members of the United Nations (UN) and the World Health Organization (WHO) [11]. Tuberculosis is a chronic communicable infectious disease caused by *Mycobacterium tuberculosis* [12,13], which mostly affects the lungs, alongside other parts of the body [14,15], and is a major cause of morbidity and fatalities [16,17]. The disease spreads through the air when infected people cough, sneeze, or spit [18]. South Africa continues to be among the highest-burdened countries in all three WHO-defined TB groupings, including TB, MDR-TB, and TB and HIV co-infection cases [1]. Over 322,000 new cases of TB are reported per year, out of which 4∙4% are DR-TB [19]. The number of MDR-TB cases keeps rising while the number of drug-susceptible TB cases appears to be declining, and this has made TB control and eradication challenging [20,21]. TB is one of the leading infectious diseases afflicting global health, and its drug-resistant form is burdensome to health systems, particularly in poorly resourced settings [22]. The Oliver Reginald (O.R.) Tambo District Municipality, located in the Eastern Cape Province of South Africa, is one of the areas of concern in this region due to its high burden of TB and DR-TB cases [23,24]. According to O.R. Tambo District Municipality population data published in 2019 by Statistics S.A, the total population was 1,514,306 people, with a gender distribution of 53.3% females and 46.7% males [25]. The population at the time was predominantly young, with 52% under the age of 20, 36% between 20 and 49 years old, 5% aged 50 to 59, and a smaller percentage over 60 years [25]. This youthful demographic presents both opportunities and challenges for the region, particularly in terms of education, employment, and healthcare needs. A significant portion of the population, 66.5%, lives in poverty, highlighting the economic challenges faced by residents of the O.R. Tambo District. The unemployment rate was reported at 37.71% in 2018, indicating a critical need for job creation and economic development initiatives. Within the employment sector, 33.4% of the workforce was employed in community services, reflecting the importance of this sector in providing essential services to the population [25]. In 2019, the region had 354,168 households, with 57.2% of these households headed by women. Notably, 14,313 households were headed by children aged 15–19, raising concerns about the implications for youth welfare and education. The healthcare infrastructure includes 137 clinics and 16 hospitals, which are vital for addressing the health needs of the population [25]. The demographic and socio-economic profile of the O.R. Tambo District Municipality reveals significant challenges related to poverty, unemployment, and healthcare access. Understanding these dynamics is essential for developing focused interventions to improve the well-being of the population.

The incidence of TB in the population shows varying levels of drug resistance. There were 5.22 cases of RR-TB per 100,000 people. MDR-TB was slightly higher, with 6.14 cases per 100,000 population. Pre-XDR-TB was recorded at a rate of 0.46 cases per 100,000, while XDR-TB was much rarer, with 0.07 cases per 100,000 population. Additionally, INHR-TB occurred at a rate of 0.13 cases per 100,000 people. MDR-TB had the highest incidence rate, slightly surpassing RR-TB. The more severe and resistant forms of TB, including Pre-XDR-TB, XDR-TB, and INHR-TB, had significantly lower incidence rates. Although these forms are rarer, they present more complex treatment challenges.

This study focused on identifying and analyzing the geographic hotspots of DR-TB within the O.R. Tambo District Municipality. The significance of this study lies in its potential to inform evidence-based public health strategies aimed at controlling DR-TB in one of the most affected regions of South Africa. By mapping the distribution of DR-TB cases, this study provides critical insights into the epidemiological patterns of the disease, which are essential for developing specific interventions. Furthermore, this research contributes to the broader understanding of the factors driving DR-TB transmission in high-burden settings, with implications for both local and national TB control programs.

## 2. Materials and Methods

### 2.1. Study Design

This was a retrospective cross-sectional cohort study. Patients’ demographic, clinical, and treatment outcome data were extracted from patient clinic files in five selected healthcare facilities of the O.R. Tambo District Municipality. The inclusion criteria were patients with DR-TB between 2018 and 2020.

### 2.2. Data Collection and Analysis

Data were collected from patients with DR-TB treated between January 2018 and December 2020 in four DR-TB decentralized clinics of the O.R. Tambo District Municipality and one referral hospital in the Eastern Cape Province. Census data for the O.R. Tambo District Municipality were obtained from Statistics South Africa. We collected global positioning system (GPS) locations of patients’ homes and diagnosing facilities. Proportion (%) was calculated when the data were categorical while the mean or median (±standard deviation) was computed when the data were continuous. We compared the sociodemographic characteristics of patients with DR-TB to the underlying O.R. Tambo general population. Student’s *t-test* was performed to assess differences between two means and the ANOVA between groups. Either the Chi-square test with and without trend or Fischer’s exact test was used to test the degree of association of categorical variables. DBSCAN (density-based spatial clustering of applications with noise), a density-based clustering technique which identifies clusters of points which are closely packed together while labeling points in sparse regions as noise (outliers), was used, combined with geospatial data (latitude and longitude), to determine clusters of DR-TB cases. Spatial autocorrelation was used to perform the hotspot analysis (Getis-Ord Gi), a spatial statistical method to determine statistically significant clusters of high or low values. Two predictive models (Linear Regression and Random Forest) were used over time to estimate DR-TB cases in hotspot areas. Linear Regression is a parametric method assuming a linear relationship, making it easy to interpret but limited in capturing complex patterns. Random Forest is a non-parametric, ensemble learning method that can capture more complex, non-linear relationships in the data. Each of these models was trained on the historical data and used to forecast future DR-TB cases. Python version 3.8. (Beaverton, Oregon, USA) and R version 4.1.1 (R Foundation for Statistical Computing, Vienna, Austria) software were used. A *p* < 0.05 was considered to be significant.

## 3. Results

A total of 456 patients were enrolled in this study, of which 56.1% were males and 43.9% females. The mean age (±SD) of the study population was 37.5 (±14.9) years. Considering a total population of 1,514,306 in the O.R. Tambo District Municipality during the study period, the incidence of DR-TB cases per 100,000 population, based on the provided data, was estimated to be 11.89 cases per 100,000 population. The age distribution analysis showed that DR-TB was significantly more prevalent among individuals aged 20–59 years, while those under 20 and over 60 were less affected compared to the general population in the O.R. Tambo region. Females were significantly underrepresented among patients with DR-TB, suggesting that males might be at higher risk or more likely to be diagnosed with DR-TB in this region (Table 1).

About 19.78% of patients diagnosed with DR-TB had no level of education, compared to the 10% recorded within the general population, and this difference reached statistical significance. Likewise, 56.85% of patients with DR-TB had no source of income, compared to the 40% recorded in the general population, as displayed in Table 1 above.

**Figure 1:**
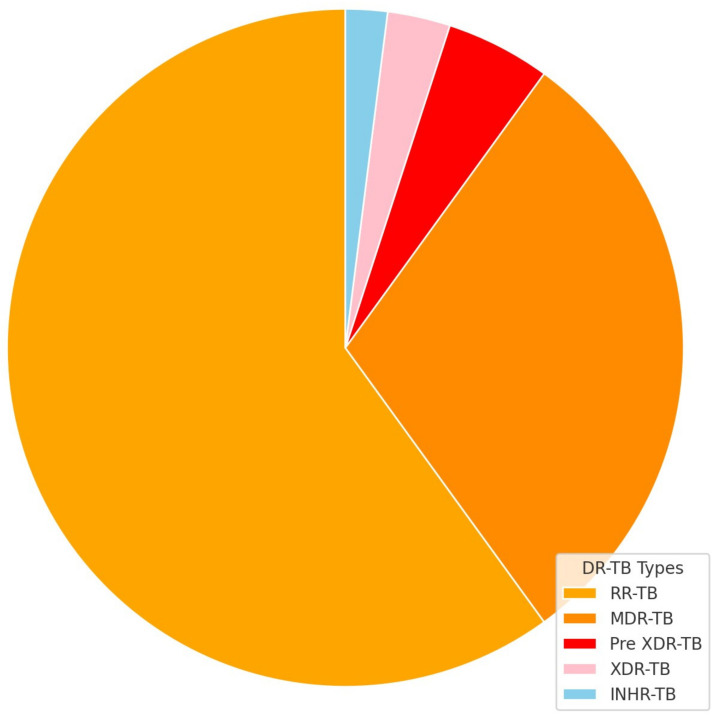
Proportion of DR-TB types within total DR-TB cases.

The distribution of DR-TB cases in Figure 1 below indicates that RR-TB accounts for the majority, with 60% of cases, followed by MDR-TB at 30%. Less prevalent forms are Pre-XDR-TB at 5%, XDR-TB at 3%, and INHR-TB at 2%. 

**Figure 2:**
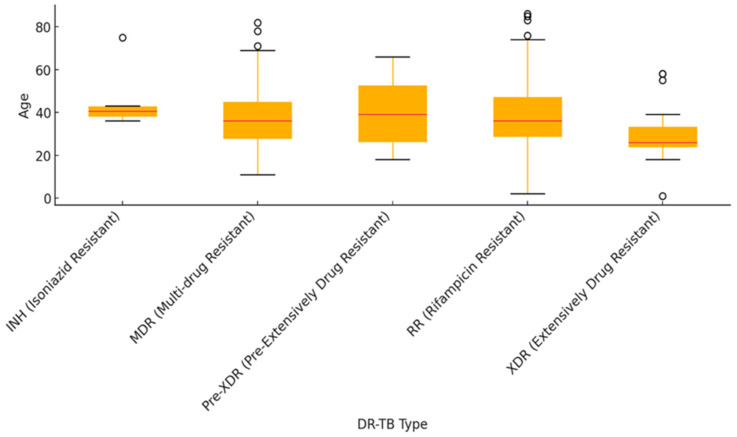
Age distribution by DR-TB type. Circles refer to outliers (data points that fall significantly outside the range of the rest of the data).

The age distribution for different DR-TB types varied significantly. MDR-TB and RR-TB showed wider age ranges with several older outliers, indicating these types affected a broader age group. In contrast, INHR-TB had the smallest variation in age. XDR-TB tended to affect younger individuals overall, though there was one notable older outlier in this group. This suggests different age-related patterns of resistance across TB types (Figure 2).

**Figure 3:**
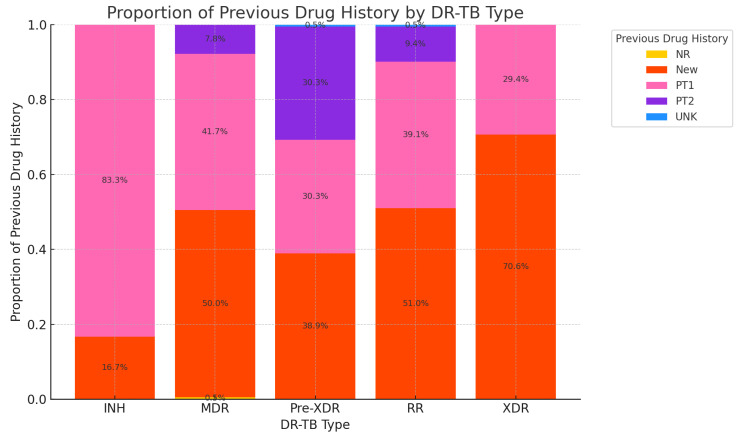
Proportion of previous drug history by DR-TB type: PT1 (previously treated once), PT2 (previously treated twice), UNK (unknown), and NR (not written).

Among INH cases, the majority (83.3%) had one PT1, while 16.7% were new patients. No cases were observed in other categories (PT2, UNK, or NR). For MDR cases, half (50%) were new patients, 41.7% had one PT1, and 7.8% had two PT2, with a minimal 0.5% recorded as NR. Pre-XDR cases were evenly distributed between new (39.1%), PT1 (30.4%), and PT2 (30.4%) categories, with no cases in NR or UNK. In RR cases, new patients dominated at 51%, followed by 39.1% in PT1 and 9.4% in PT2. A small proportion (0.5%) fell into the UNK category. XDR cases overwhelmingly consisted of new patients (70.6%), with 29.4% in PT1, and no reported cases in other categories (Figure 3).

**Figure 4:**
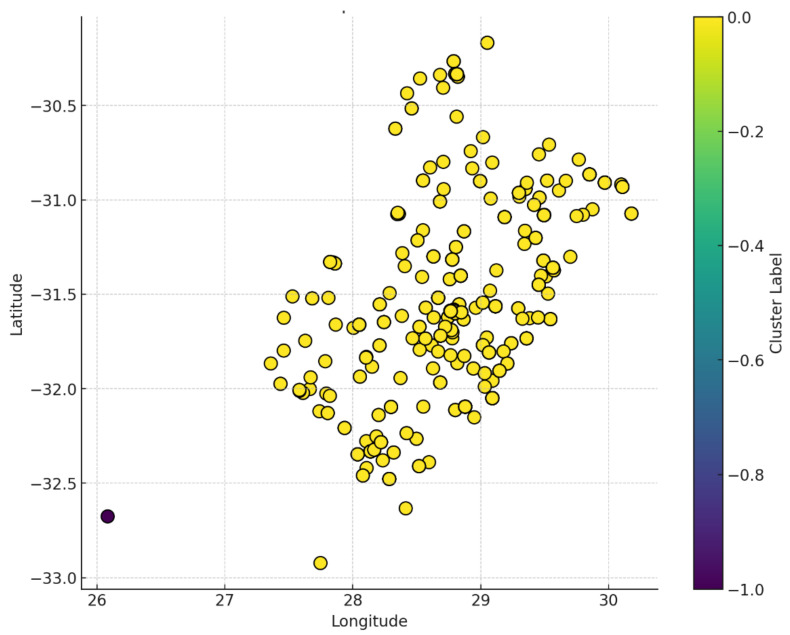
DR-TB hotspots in the O.R. Tambo District Municipality.

In Figure 4, displaying DR-TB hotspots, the color bar on the right represents the cluster labels, with a gradient ranging from dark purple to yellow. This color scale corresponds to different clusters or groups of data points, where the range from −1.0 to 0.0 likely indicates cluster identification or a measure related to the clusters, such as a silhouette score or another clustering metric. Points in the scatter plot are colored according to their cluster label, with yellow being the predominant color, suggesting that most points belong to the same cluster. A few outliers, such as the dark purple point, may belong to a different or unique cluster. Most of the data points are concentrated between longitudes of 27 to 30 and latitudes of −30.5 to −32.0. There is a distinct outlier located at approximately (26, −32.5), colored dark purple, indicating that it belongs to a different or unique cluster compared to the majority of the points. The fact that most data points are colored in yellow suggests that a large portion of the dataset belongs to a single, dominant cluster. This could imply that the majority of the data share similar characteristics, leading to a high degree of cohesion.

**Figure 5:**
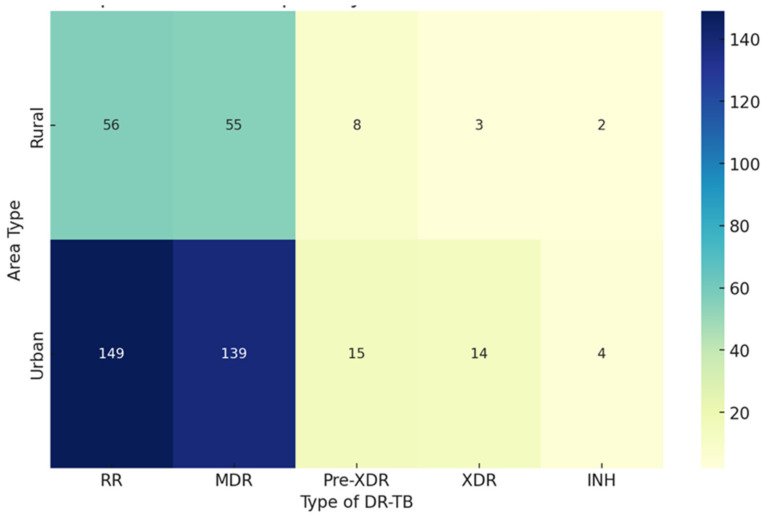
Heat map of DR-TB hotspots by urban and rural classification.

Here is the heat map visualizing DR-TB hotspots by urban and rural classification. The intensity of the color represents the number of DR-TB cases, with annotations showing the exact counts. The heat map clearly shows that DR-TB, particularly the RR and MDR types, is more prevalent in urban areas compared to rural areas. While urban areas have higher counts across all DR-TB types, rural areas show a much smaller distribution, especially for the more severe forms like Pre-XDR-TB, XDR-TB, and INH DR-TB (Figure 5).

**Figure 6:**
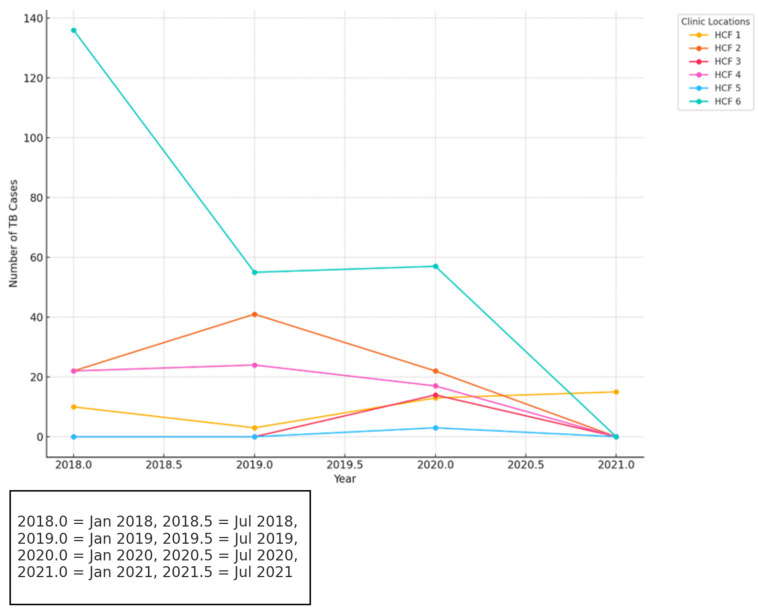
TB trends by clinic locations.

The analysis of TB case trends across clinics in Figure 6 highlights significant patterns. HCF 6 consistently reported the highest number of TB cases throughout the study period, with a sharp decline from approximately 140 cases in 2018 to nearly 0 by 2021. Meanwhile, HCF 2 and HCF 4 exhibited relatively steady declines, reflecting consistent yet moderate progress. Conversely, HCF 1 and HCF 3 demonstrated smaller fluctuations, with low initial case counts followed by increases in 2020 and 2021, necessitating closer monitoring to address potential emerging risks. By 2021, most clinics reported near-zero TB cases. These reductions may reflect disruptions in healthcare reporting, potentially influenced by external factors like the COVID-19 pandemic. Overall, HCF 6 stands out as the most significant TB hotspot, contributing the largest proportion of cases during the studied period. The ANOVA test (F = 4.33, *p* = 0.028) shows statistically significant differences in the number of TB cases among clinics (*p* < 0.05), suggesting that some clinics, such as HCF 6, experienced substantially higher case counts compared to others. This highlights the importance of targeting interventions in hotspot clinics to address the disproportionate burden. However, the t-test comparing HCF 6 to the second-highest clinic (t = 1.98, *p* = 0.173) indicates no statistically significant difference (*p* > 0.05) in case volumes between the two. While HCF 6 holds the highest total case counts, the overlap in case distributions suggests that both clinics may share similar challenges or patterns, requiring tailored interventions.

**Figure 7:**
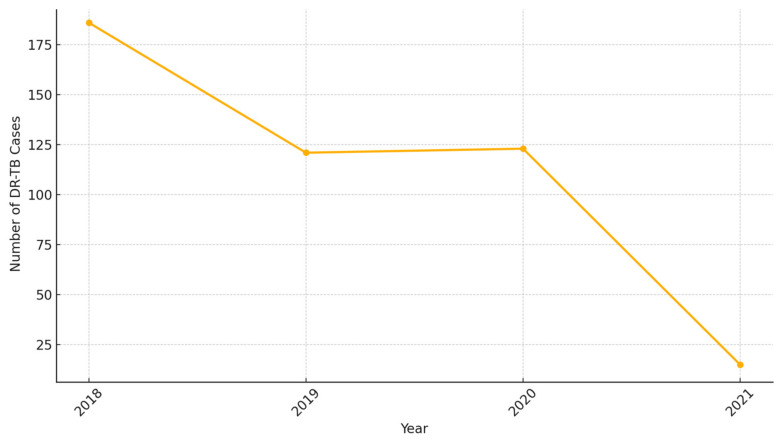
DR-TB trends over time.

Figure 7 below illustrates the temporal trends of DR-TB cases from 2018 to 2021 in hotspots. From 2018 to 2021, DR-TB cases demonstrated significant temporal variations. Between 2018 and 2019, there was a sharp decline in cases, dropping from 186 to 121, representing a ~35% reduction. The trend stabilized between 2019 and 2020, with a marginal increase from 121 to 123 cases, indicating a possible plateau in case reductions or challenges in maintaining intervention momentum. However, between 2020 and 2021, a dramatic reduction was observed, with cases plummeting from 123 to just 15 (~88% reduction).

**Figure 8:**
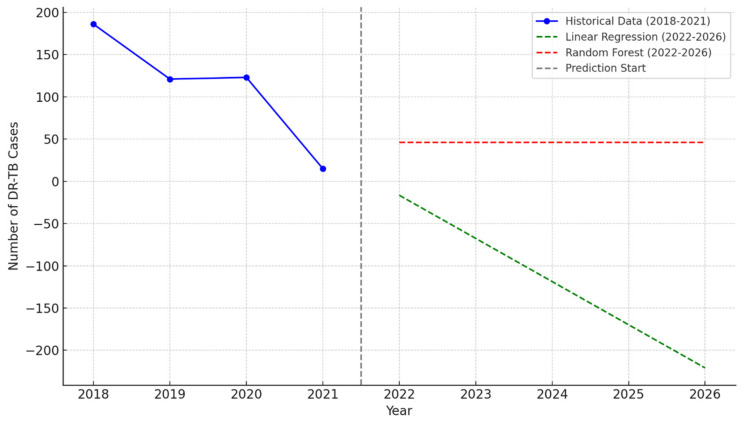
Historical and predicted DR-TB cases (2018–2026).

In Figure 8, from 2018 to 2021, historical data reveal a significant decline in DR-TB cases, with the numbers dropping steadily from 186 in 2018 to just 15 in 2021. Looking ahead, predictions for 2022 to 2026 provide differing perspectives on future trends. The Linear Regression model forecasts a continued decline in DR-TB cases, potentially reaching zero by 2026 if current trends persist, highlighting the possibility of achieving elimination. In contrast, the Random Forest model predicts stabilization, with cases plateauing at approximately 30 annually from 2022 onward, indicating that, while further improvements may be limited, the issue remains contained. These divergent forecasts underscore the importance of sustained monitoring and intervention to ensure that progress aligns with the most optimistic scenarios.

**Figure 9:**
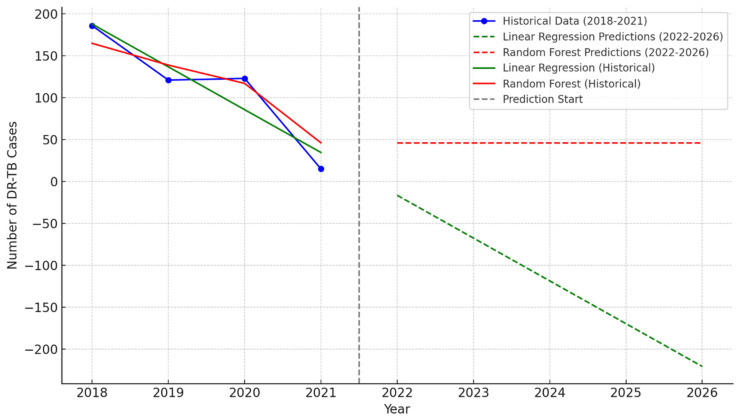
DR-TB case trends: Linear Regression versus Randon Forest.

The performance metrics for the Linear Regression and Random Forest models (Figure 9) provide valuable insights into their effectiveness in predicting DR-TB cases based on historical data (2018–2021). Both models demonstrate strong predictive capabilities, but their strengths and limitations vary. The Linear Regression model achieves an R^2^ = 0.865, indicating that it explains 86.5% of the variance in DR-TB cases. This reflects a strong fit to the data, particularly in capturing the overall downward trend. The Random Forest model performs slightly better, with an R^2^ = 0.882, capturing 88.2% of the variance. Its higher score highlights its ability to model non-linear patterns and account for variability in the data. The Linear Regression model has a mean squared error (MSE) of 507.175, showing larger average squared differences between predictions and actual cases, especially in years with sharp declines (e.g., 2020 and 2021). The Random Forest model, with a lower MSE of 443.226, handles year-to-year variability more effectively, producing smaller squared errors and better accuracy in reflecting data fluctuations. Linear Regression yields a mean absolute error (MAE) of 18.65, indicating that predictions deviate from actual cases by approximately 18.65 cases on average. While the model captures broad trends, it struggles with precision in years with sharp changes. Random Forest has a slightly higher MAE of 19.03, reflecting marginally less accuracy in capturing smaller fluctuations, likely due to its smoothing effect. The Random Forest model outperforms Linear Regression in capturing variability and non-linear trends, as evidenced by its higher R^2^ and lower MSE. However, Linear Regression remains effective in modeling overall trends and provides a more straightforward interpretation of case reductions. Both models offer complementary insights, with Random Forest being better suited for capturing complex patterns and Linear Regression providing simpler, trend-based forecasts. Together, they offer robust frameworks for analyzing DR-TB trends and informing intervention strategies.

## 4. Discussion

Our investigation is particularly timely given the increasing global focus on eliminating TB as a public health threat. DR-TB is a major challenge to national TB control pro-grams in developing countries and a leading cause of death in South Africa [26,27]. As South Africa strives to achieve its TB control goals, understanding the spatial dynamics of DR-TB in high-burden areas like the O.R. Tambo District Municipality is essential for working on solutions that can effectively address the unique challenges posed by DR-TB strains. The investigation into the dynamics of DR-TB in the O.R. Tambo District Municipality reveals critical insights into the factors influencing its transmission and control. These findings are particularly significant given the global focus on eliminating TB as a public health threat and addressing the challenges posed by DR-TB strains, especially in high-burden areas like South Africa. As DR-TB remains a major challenge to national TB control programs and a leading cause of death in South Africa, this study provides timely information for developing effective, localized interventions.

This study highlights a disproportionate burden of DR-TB among individuals aged 20–59 years, who are at the greatest risk due to a combination of social, economic, and health factors [28,29]. This age group is the most economically active, increasing their likelihood of exposure to TB in crowded workplaces, public spaces, and social gatherings [30,31]. Additionally, individuals in this demographic often face challenges such as poor nutrition, lack of healthcare access, and delayed diagnoses, exacerbating their vulnerability to TB and the development of drug resistance [32].

HIV co-infection is another significant contributor, compromising immune systems and increasing susceptibility to DR-TB [33]. Stress, stigma, and financial barriers further hinder treatment adherence, contributing to the emergence of resistant strains [34]. Conversely, younger populations (under 20 years) benefit from vaccinations and lower exposure rates, while older individuals (over 60 years) have reduced social interactions, lowering their exposure risk despite age-related vulnerabilities [35]. Targeted interventions for the 20–59 age group, combined with preventative strategies for younger and older populations, are essential for controlling DR-TB transmission.

Our study reveals that traditional gender roles can influence who is most exposed to TB. Females constituted 44.04% of the total. In contrast, the general population of the O.R. Tambo District, which had a total population of 1,514,306, showed higher female representation, at 53.3%. This indicates that the proportion of females among patients with DR-TB is lower than that of the general population by approximately 9.26%. The 95% confidence interval (CI) of −9.30 to −9.21 suggests that the observed difference in gender distribution is statistically significant, as well as the *p*-value of 0.000035. It is well recognized in the literature that greater rates of TB are found in males than in females [36,37]. Males are often more exposed to environments where TB transmission is more likely, such as workplaces, public transportation, and social settings like bars or community gatherings. The dynamics of TB transmission suggest that community settings, where males often congregate, can be significant contributors to the spread of the disease. The risk is compounded in environments with poor ventilation and high occupancy [38,39]. These settings often have higher TB transmission rates, especially in densely populated or high-prevalence areas [40,41,42]. Additionally, higher rates of smoking and substance abuse among men weaken their immune systems, heightening their risk of contracting and developing DR-TB [43,44]. Women, however, tend to demonstrate better adherence to TB treatment, possibly due to more frequent healthcare interactions related to reproductive health or childcare responsibilities [45,46,47]. This difference in treatment adherence highlights the need for gender-sensitive approaches in addressing DR-TB, ensuring that both men and women have access to timely diagnosis and effective treatment.

Poverty emerges as a critical risk factor for DR-TB, linking overcrowded and poorly ventilated living conditions to higher transmission rates [48,49,50]. Malnutrition, common in impoverished populations, weakens immune systems, increasing susceptibility to TB and hindering recovery [51,52,53]. This study highlights that malnourished individuals are three times more likely to develop TB compared to their well-nourished counterparts. Financial constraints often prevent timely medical care, delaying diagnoses and increasing the risk of drug resistance [54]. Additionally, socio-economic barriers such as transportation costs, loss of income, and stigma deter individuals from completing TB treatment, further contributing to the emergence of DR-TB strains [55]. Education plays a significant role in influencing TB prevention and treatment adherence [49]. Individuals with no formal education are less likely to recognize TB symptoms or understand the importance of completing treatment regimens, increasing their risk of developing drug resistance [56]. Women from lower socio-economic backgrounds face additional barriers, such as stigma and financial dependence, which hinder their ability to seek timely care [57,58]. Public health efforts must prioritize educational campaigns to raise awareness about DR-TB, particularly in high-risk, low-education populations.

The majority of RR-TB and MDR-TB cases are classified as “new”, indicating that resistance can develop even in individuals without prior TB treatment. This underscores the need for strong initial treatment protocols to prevent the emergence of resistance. Pre-XDR-TB and extensively XDR-TB cases are more prevalent among individuals with prior treatment histories, highlighting the importance of monitoring and managing treatment regimens to prevent resistance escalation.

The prevalence of new patients among XDR (70.6%), RR (51%), and MDR (50%) cases suggests that a significant share of resistance emerges in previously untreated patients [59], particularly in the most severe cases (XDR). PT1 is most common in INH (83.3%) and substantial in MDR (41.7%) and RR (39.1%), indicating that one prior treatment is closely associated with resistance development [60]. XDR-TB presents a slightly different distribution, with a higher proportion of cases classified as PT1 and PT2 compared to RR-TB and MDR-TB [60]. This suggests that XDR-TB is more likely to occur in individuals with a history of inadequate treatment, reinforcing the need for careful monitoring and the management of TB treatment regimens to prevent the emergence of such resistant strains [61]. The smaller proportion of “new” cases in the XDR category further indicates that prior treatment experiences significantly influence the likelihood of developing this severe form of drug resistance [60]. PT2 cases are notable in Pre-XDR (30.4%) and RR (9.4%), hinting at an increased risk of severe resistance with repeated treatments. INHR-TB exhibits a simpler distribution, with the majority of cases classified as “new” and a significant portion in the PT1 category, while PT2 and unknown histories are less prevalent [62]. This suggests that INHR-TB may not be as heavily influenced by previous treatment failures as other forms of DR-TB, although the risk of developing multidrug resistance remains a concern [63]. The minimal presence of NR and UNK categories reflects good documentation of patient histories. The findings underscore the importance of monitoring and effectively treating patients with a history of TB to prevent the escalation of drug resistance. The high proportion of new patients in XDR and RR cases highlights the need for robust interventions to curb resistance development in patients who have TB for the first time. This study also reveals that incomplete or inadequate treatment significantly contributes to the development of resistant strains. Factors such as interruptions in treatment, inappropriate drug regimens, and poor adherence leave some TB bacteria alive, allowing them to develop resistance. Focused public health interventions targeting these treatment gaps are essential for preventing the progression of resistance.

The median age of DR-TB cases is consistently around 40 years, with the majority of cases affecting individuals aged 30–50. These demographic faces higher exposure risks due to their active workforce participation and social environments [7]. The intersection of chronic conditions such as HIV and diabetes further heightens their vulnerability to DR-TB, complicating treatment and management strategies [57]. These findings emphasize the need for focused interventions targeting middle-aged individuals, ensuring adequate support for both prevention and treatment adherence. MDR, Pre-XDR, and RR TB types affect a broader age range due to varied transmission patterns and diverse risk factors, allowing these forms of DR-TB to impact individuals across different age groups. MDR-TB, Pre-XDR-TB, and RR-TB exhibit varied transmission patterns that allow them to affect individuals across different age groups. For instance, studies have shown that MDR-TB can be transmitted in both community and healthcare settings, leading to infections among younger populations and older adults alike [56]. The WHO reports that the incidence of MDR-TB is rising among younger individuals, particularly in regions with high TB prevalence [6,57]. INH and XDR cases were more concentrated around the median age. Conversely, INHR-TB and XDR-TB cases being more concentrated around the median age might indicate that these forms of resistance are more likely to develop in middle-aged individuals due to the specific treatment histories and exposure risks associated with this age group. This may lead to the development of drug resistance and inadequate treatment adherence or incomplete courses of therapy, and exposure to suboptimal treatment regimens can contribute to the emergence of INHR-TB and XDR-TB [57].

This study highlights significant spatial clustering of DR-TB cases in the O.R. Tambo region, particularly in high-density urban areas. In densely populated areas, close living conditions and increased social interactions can contribute to higher rates of TB infection [56]. Overcrowding, poverty, and limited healthcare access create conditions conducive to TB transmission, with stigmatization and inadequate health literacy exacerbating the problem [56,57,64]. In many low-income settings, healthcare facilities may be scarce, poorly equipped, or lacking in trained personnel, leading to delays in diagnosis and treatment initiation [56]. A study found that individuals in areas with limited healthcare access were more likely to experience prolonged illness and increased rates of drug resistance due to inadequate treatment [57]. Identifying these hotspots is critical for resource allocation and targeted interventions. For instance, areas with high poverty indicators, such as low educational attainment and unemployment rates, show elevated DR-TB prevalence. Addressing these clusters through tailored public health strategies can significantly reduce transmission.

The introduction of revised TB treatment regimens in 2018 positively impacted DR-TB management, with shorter and more effective regimens improving patient adherence and reducing treatment failure [65]. For example, in South Africa, 66% of patients with drug-resistant TB were placed on WHO-recommended shorter regimens by 2022, contributing to better outcomes. However, the COVID-19 pandemic disrupted TB control efforts, delaying diagnoses and reducing treatment adherence [66]. Social distancing measures and reduced mobility temporarily decreased TB transmission, but the long-term effects of these disruptions necessitate ongoing attention to prevent setbacks in TB control. There was a decline in TB notifications during periods of strict lockdowns, suggesting that reduced mobility played a role in limiting transmission [57]. The pandemic altered health-seeking behaviors, with many individuals hesitant to seek care due to fears of COVID-19 exposure. This led to a decline in TB testing and treatment adherence, which could have long-term implications for TB control efforts [67]. The COVID-19 pandemic likely impacted TB case reporting and diagnosis, potentially leading to a decrease in reported cases [68]. With fewer people interacting in crowded settings, the risk of airborne diseases like TB decreased. Data from various studies show a consistent decline in both drug-sensitive and drug-resistant TB cases in recent years. For instance, a study high-lighted a sharp drop in DR-TB cases during the COVID-19 pandemic, emphasizing the impact of less transmission due to the protocols which had been put into place [68]. However, this also resulted in delays in diagnosis and treatment for many existing patients with TB [69].

This study utilized predictive models to forecast DR-TB trends, revealing contrasting outcomes. The Linear Regression model predicted a continued decline in DR-TB cases, projecting complete elimination by 2026. While optimistic, this model assumed a linear trend and may have overestimated progress. In contrast, the Random Forest model anticipated stabilization, suggesting that, without enhanced interventions, case reductions may plateau. These predictions emphasize the need for continued monitoring and adaptive public health strategies to sustain progress.

When using historical data (2018–2021) and predictions (2022–2026) generated from Linear Regression and Random Forest models for assessing DR-TB case trends and model performance, we analyzed trends to assess progress and project future outcomes. The historical data revealed a sharp decline in DR-TB cases, from 186 cases in 2018 to just 15 in 2021. This reduction suggests the effectiveness of interventions, including enhanced diagnostic strategies, improved access to treatment, and strengthened public health initiatives. Similar outcomes have been observed in global settings where MDR-TB programs were scaled up [1]. However, challenges like healthcare disruptions, particularly during the COVID-19 pandemic, may have contributed to underreporting in later years [70].

Regarding model predictions (2022–2026), the Linear Regression model relied on the maintenance of current trends, without interruptions. This model provides a simple, interpretable framework for understanding trends. It aligns with studies emphasizing steady case reduction through intensified intervention programs [71]. The projection of negative case counts highlights the model’s oversimplification of real-world complexities, such as plateau effects or resource constraints in high-burden areas. The Random Forest model predicted stabilization to around 30–50 cases annually by 2022, reflecting a more conservative and realistic scenario. This model accounts for non-linear patterns and variability, aligning with findings that DR-TB control often reaches a plateau due to incomplete treatment adherence or delayed detection [72].

Regarding model performance metrics, Linear Regression achieved an R^2^ = 0.865, and Random Forest scored slightly higher, at R^2^ = 0.882. These values indicate strong fits for both models, with Random Forest better capturing variability in the data. Random Forest outperformed Linear Regression in terms of MSE (443.226 vs. 507.175), reflecting its ability to reduce large prediction errors. Both models demonstrated comparable MAE values (Linear Regression: 18.65; Random Forest: 19.03), indicating similar average deviations from actual data. These metrics align with findings from modeling studies that highlight the suitability of Random Forest for complex, non-linear relationships in epidemiological data [73].

## 5. Conclusions

This study underscores the complex socio-economic and demographic factors contributing to the spread of DR-TB in the O.R. Tambo District Municipality. The findings highlight the disproportionate impact of DR-TB on individuals aged 20–59 years, with a significant overrepresentation of males in the DR-TB population. Key factors such as poverty, inadequate healthcare access, and malnutrition exacerbate the risk of developing DR-TB, especially in high-density, disadvantaged areas. Focused interventions, including improved healthcare access, educational campaigns, and nutritional support, are essential for reducing the transmission of DR-TB in these high-risk groups. Additionally, strategies to improve treatment adherence and address gender-specific risks are critical to combatting the spread of DR-TB. As South Africa strives to meet its TB control goals, these findings emphasize the need for holistic approaches that address the socio-economic determinants of DR-TB to prevent the emergence of more severe drug-resistant strains.

The Random Forest model outperformed Linear Regression in capturing variability and non-linear trends, as evidenced by its higher R^2^ and lower MSE. However, Linear Regression remained effective in modeling overall trends and provided a more straightforward interpretation of case reductions. Both models offer complementary insights, with Random Forest being better suited for capturing complex patterns and Linear Regression providing simpler, trend-based forecasts. Together, they offer robust frameworks for analyzing DR-TB trends and informing intervention strategies.

## Figures and Tables

**Table 1:** Baseline characteristics of patients with DR-TB in the O.R. Tambo District compared to the general population’s characteristics.

Characteristics of Interest	Enrolled Patients with DR-TB (n = 456) (%)	O.R. Tambo Population (n = 1 514 306) %	Difference %	[95% CI]	*p*-Value
Demographic characteristics					
Age (years)					
<20	30.0	52.0	−22.0	[−22.04; −21.96]	<0.0001
20–49	50.0	36.0	14.0	[13.96; 14.04]	<0.0001
50–59	15.0	5.0	10.0	[9.97; 10.03]	<0.0001
>60	5.0	7.0	−2.0	[−2.02; −1.98]	0.0597
Gender					
Female	44.04	53.3	−9.26	[−9.30; −9.21]	0.000035
Level of education					
No Education	19.78	10	+9.78	[9.75%, 9.81%]	<0.0001
Primary Education	22.92	30	−7.08	[−7.12%, −7.04%]	0.00033
Secondary Education	47.87	50	−2.13	[−2.17%, −2.09%]	0.3407
Tertiary Education	9.21	10	−0.79	[−0.82%, −0.76%]	0.5488
Income category					
No Income	56.85	40	+16.85	[16.81%, 16.89%]	<0.0001
Salary or Wages	19.15	25	−5.85	[−5.89%, −5.81%]	0.0016
Casual	15.96	20	−4.04	[−4.07%, −4.01%]	0.0187
UIF	1.54	5	−3.46	[−3.48%, −3.44%]	0.00036
Disability Grant	3.08	5	−1.92	[−1.94%, −1.90%]	0.0292

## Data Availability

Data can be requested from the corresponding author.
